# Altered Aortic Hemodynamics and Relative Pressure in Patients with Dilated Cardiomyopathy

**DOI:** 10.1007/s12265-021-10181-1

**Published:** 2021-12-09

**Authors:** David Marlevi, Jorge Mariscal-Harana, Nicholas S. Burris, Julio Sotelo, Bram Ruijsink, Myrianthi Hadjicharalambous, Liya Asner, Eva Sammut, Radomir Chabiniok, Sergio Uribe, Reidar Winter, Pablo Lamata, Jordi Alastruey, David Nordsletten

**Affiliations:** 1 Institute for Medical Engineering and Science, Massachusetts Institute of Technology, Cambridge, MA USA; 2 Department of Biomedical Engineering and Health Systems, KTH Royal Institute of Technology, Huddinge, Sweden; 3 Department of Clinical Sciences, Karolinska Institutet, Danderyd, Sweden; 4 School of Biomedical Engineering and Imaging Sciences, King’s College London, London, UK; 5 Department of Radiology, University of Michigan, Ann Arbor, MI USA; 6 School of Biomedical Engineering, Universidad de Valparaíso, Valparaíso, Chile; 7 Biomedical Imaging Center, Pontificia Universidad Catolica de Chile, Santiago, Chile; 8 Millennium Nucleus in Cardiovascular Magnetic Resonance, Santiago, Cardio MR Chile; 9 Department of Mechanical and Manufacturing Engineering, University of Cyprus, Nicosia, Cyprus; 10 Faculty of Health Science, Bristol Heart Institute and Translational Biomedical Research Centre, University of Bristol, Bristol, UK; 11 Inria, Palaiseau, France; 12 LMS, Ecole Polytechnique, CNRS, Institut Polytechnique de Paris, Paris, France; 13 Department of Mathematics, Faculty of Nuclear Sciences and Physical Engineering, Czech Technical University in Prague, , Prague, Czech Republic; 14 Department of Radiology, School of Medicine, Pontifica Universidad Católica de Chile, Santiago, Chile; 15 World-Class Research Center “Digital Biodesign and Personlized Healthcare”, Sechenov University, Moscow, Russia; 16 Department of Cardiac Surgery and Biomedical Engineering, University of Michigan, Plymouth Rd, Ann Arbor, MI 48109 USA

**Keywords:** Dilated cardiomyopathy, Aortic hemodynamics, Aortic relative pressure, Aortic stiffness, 4D flow MRI

## Abstract

Ventricular-vascular interaction is central in the adaptation to cardiovascular disease. However, cardiomyopathy patients are predominantly monitored using cardiac biomarkers. The aim of this study is therefore to explore aortic function in dilated cardiomyopathy (DCM). Fourteen idiopathic DCM patients and 16 controls underwent cardiac magnetic resonance imaging, with aortic relative pressure derived using physics-based image processing and a virtual cohort utilized to assess the impact of cardiovascular properties on aortic behaviour. Subjects with reduced left ventricular systolic function had significantly reduced aortic relative pressure, increased aortic stiffness, and significantly delayed time-to-pressure peak duration. From the virtual cohort, aortic stiffness and aortic volumetric size were identified as key determinants of aortic relative pressure. As such, this study shows how advanced flow imaging and aortic hemodynamic evaluation could provide novel insights into the manifestation of DCM, with signs of both altered aortic structure and function derived in DCM using our proposed imaging protocol.

**Graphic Abstractr:**

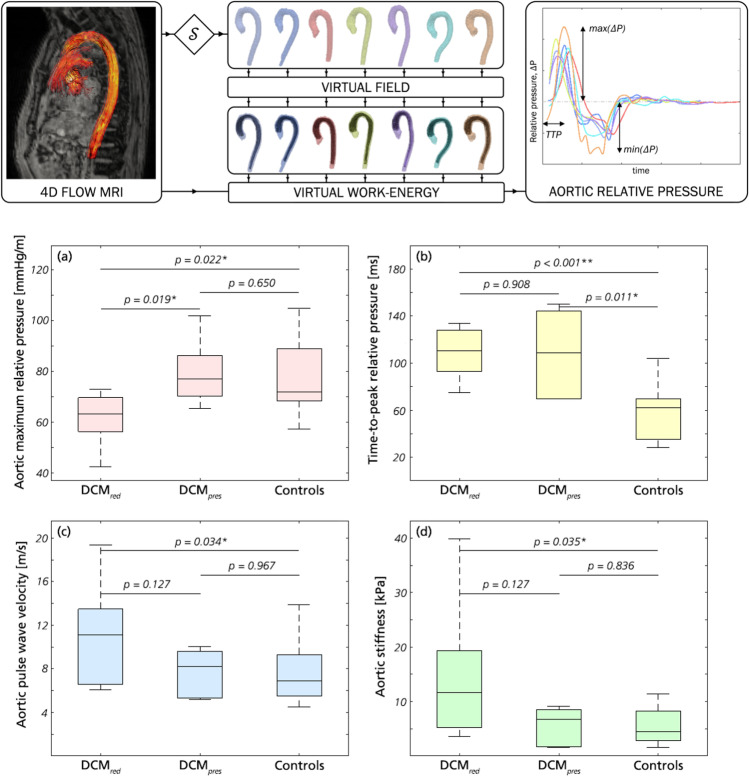

**Supplementary Information:**

The online version contains supplementary material available at 10.1007/s12265-021-10181-1.

## Introduction

The heart and the vasculature are inherently coupled, and remodelling mechanisms on one side is commonly attributed to pathological manifestation on the other. Arterial and ventricular stiffening has been observed in heart failure patients with preserved ejection fraction [6]. Pulse wave velocity and augmentation index—arterial measures related to vascular stiffness—have both been correlated to left ventricular (LV) systolic dysfunction [43, 48]. Vascular hemodynamics have also been closely coupled to ventricular function, with aortic relative pressure—the change in blood pressure over aortic segments—being linked to aortic capacitance/stiffness, LV remodelling [7], aortopathies (e.g. coarctation, aneurysm, or dissection) [28], and even hypertrophic outflow tract obstruction [21]. Despite this, arterial function is seldom studied in cardiomyopathy patients. Instead, cardiomyopathy is typically seen as a “heart-only” disease, with diagnosis and intervention guided primarily using measures of cardiac function.

Despite clear anatomical and functional phenotypes, dilated cardiomyopathy (DCM) is commonly idiopathic with associated poor prognosis [13]. Refined prognostic and diagnostic biomarkers are thus merited, and herein, ventricular-vascular interaction has been hypothesized as a key component in understanding disease progression. An increased aortic stiffness has been identified in DCM [5], and vascular hemodynamics have been linked to disease development, with systemic hypertension correlated to DCM mortality [1]. Interestingly, the prognosis following cardiac resynchronization of heart failure patients (including DCMs) [51] and the ability to induce left ventricular reverse remodelling (experienced in up to 40% of all DCM patients [36]) have been related to arterial behaviour, highlighting the importance of the vasculature.

To date, the clinical assessment of cardiovascular function has been largely based on medical imaging. In addition to diagnosing DCM by means of LV size and function, myocardial tissue characterization by cardiac magnetic resonance imaging (MRI) (using T1 or T2 mapping) is being increasingly used in clinical assessment [19]. To this, phase-contrast (PC-) MRI methods such as 2D or 4D flow MRI [16] now permit comprehensive evaluation of blood flow in the cardiovascular system. 4D flow MRI in particular has uncovered altered diastolic ventricular flow routes in DCM [17], quantified aortic relative pressure in association with ventricular remodelling [7], and has enabled accurate estimation of relative pressure in vivo [14, 34]. MRI flow imaging has been suggested as a possible tool for assessing ventricular-vascular behaviour in chronic heart failure [11] and right ventricular pulmonary coupling [29]. Thus, 4D flow MRI can provide a more comprehensive evaluation of cardiac and vascular behaviour and may allow for refined understanding of ventricular-vascular interaction in DCM patients.

Therefore, the objective of this study was to examine aortic hemodynamics in a clinical cohort of idiopathic DCM patients with reduced and preserved LV systolic function, using non-invasive 4D flow MRI, to better understand the relationship between vascular alterations and cardiac disease. In particular, aortic relative pressure—directly derived from the 4D flow MRI data—was chosen to represent the aortic hemodynamic state, based on the fact that relative pressure has been shown to describe both cardiac function and reservoir status in vivo [7, 21, 28, 32]. Furthermore, given the challenges of studying isolated effects of arterial and ventricular perturbations in vivo due to their physiologic interdependence, we developed a computational *virtual* cohort [49] (using a model which simulates blood flow in the larger systemic arteries) to understand the impact of individual cardiovascular properties on aortic relative pressure. Using this combination of 4D flow MRI and computational cardiovascular simulations techniques, we aimed to map aortic and cardiac hemodynamic function and explore possible signs of ventricular-vascular interplay in DCM.

## Methods

**Fig. 1:**
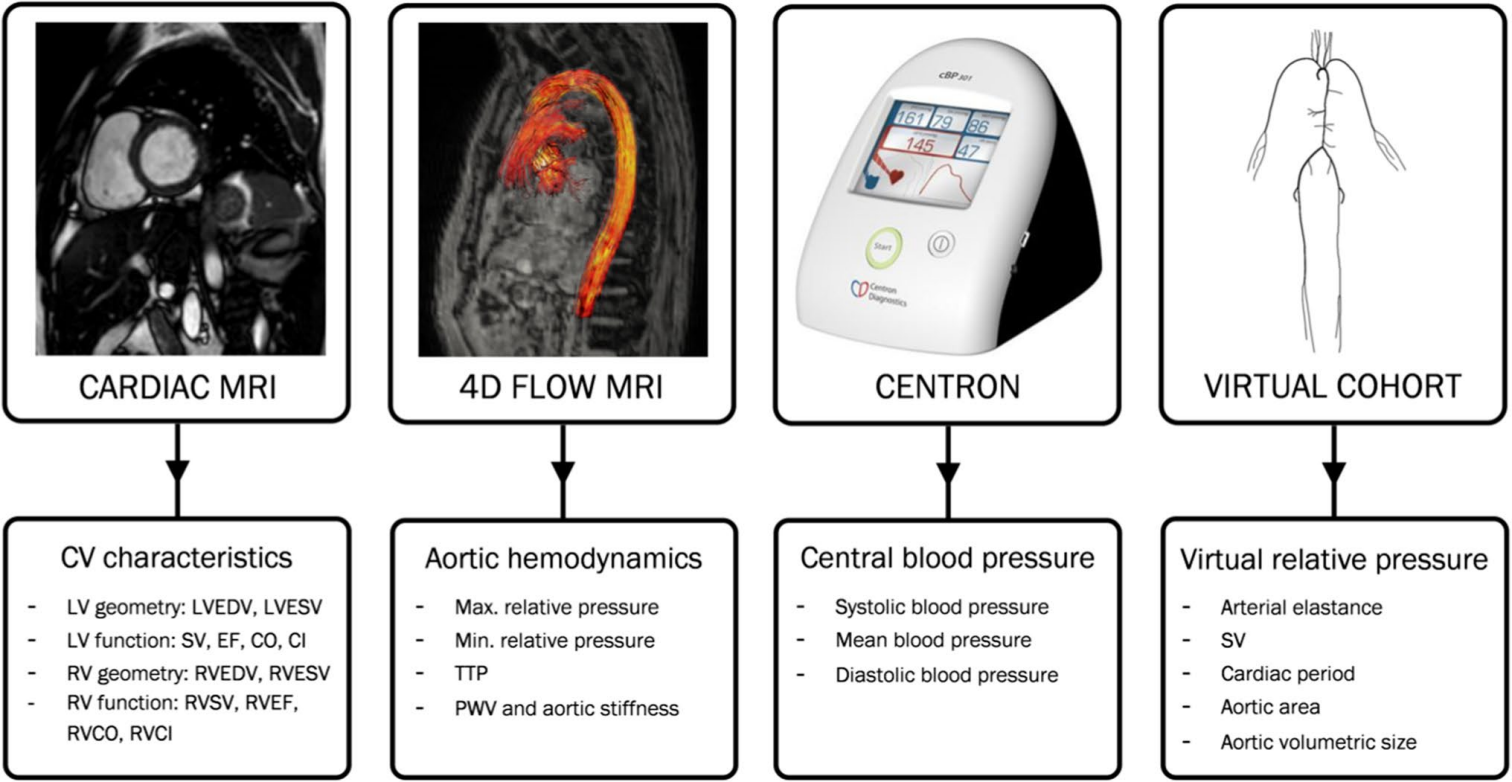
Overview of assessed study metrics. Overview of the assessed metrics of the study, with cardiovascular (CV) characteristics given by cardiac MRI, aortic hemodynamics from 4D flow MRI, and central blood pressure from sphygmomanometer measures. The isolated influence of listed cardiovascular properties on aortic relative pressure is determined using a computational virtual cohort. LV, left ventricle. RV, right ventricle. EDV, end-diastolic volume. ESV, end-systolic volume. SV, stroke volume. EF, ejection fraction. CO, cardiac output. CI, cardiac index. PWV, pulse wave velocity. TTP, time-to-peak relative pressure

Figure 1 shows an overview of all utilized data sources. Additionally, extracted quantities are specified, showing the spectrum of cardiac and aortic parameters analysed.

### Study Population

In this retrospective study, 14 patients and 16 healthy controls were included. Adult subjects were recruited at the St Thomas’ Hospital, King’s College London, through the British Heart Foundation Integrated Mathematical Modelling and Imaging for Dilated Cardiomyopathy (BHF-IMMI) project, with data acquired during March 2013–April 2014. The inclusion criteria for the patient cohort were patients aged > 18 years and diagnosed with non-ischemic symptomatic DCM-related heart failure (NYHA class III, including echocardiographic ejection fraction (EF) = 35–45% at recruitment) which was deemed idiopathic in nature after clinical evaluation. The exclusion criteria were known airway disease, pregnancy, renal or hepatic impairment, previous history of angina or cardiac arrhythmias for which continuous administration of beta blockers was deemed necessary, as well as contraindications to MRI. In addition to DCM patients, healthy control subjects without any known cardiovascular, pulmonary, renal, hepatic, diabetic, or other systemic diseases were recruited from the BHF-IMMI project. Assessment of potential valvular disease was not part of the inclusion/exclusion protocol.

Patient treatment followed guideline medical management for heart failure, specifically including beta blocker administration. At the time of MRI, a subgroup of DCM patients presented with preserved systolic LV function following medical therapy. Consequently, the DCM cohort was divided into two subgroups: subjects with reduced LV systolic function (DCM_*red*_, LV ejection fraction (EF) < 50%, *n* = 9) and subjects with preserved LV systolic function (DCM_*pres*_, EF ≥ 50%, *n* = 5). Prior to data collection, the entire patient cohort had their beta blocker treatment discontinued for 48 h to examine native cardiovascular function.

All subjects provided informed consent, with data collection approved by the Regional Ethics Committee, South East London, UK (REC, 12/LO/1456). Subject demographics are shown in Table 1.

**Table 1:**
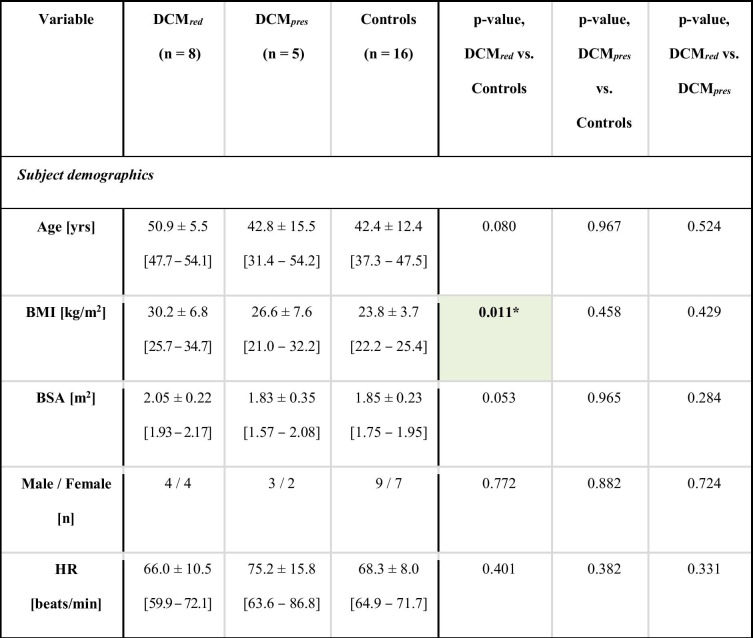
Key subject demographics. Key subject demographics for the DCM_*red*_, DCM_*pres*_, and control groups, respectively. Intragroup *p*-values are reported with significant differences indicated by * (*p* < 0.05), ** (*p* < 0.01), or *** (*p* < 0.001), as well as with the colour coding. 90% confidence intervals are given in brackets.

### Imaging, Data Collection, and Post-Processing

#### Cardiac Functional Imaging

MRI was performed at 1.5 T (Philips ACHIEVA) using a 32-channel cardiac coil. Cardiac function and characteristics were assessed using cine steady-state-free precession (SSFP) MRI of stacked short axis and three long axis view planes. Left and right ventricular end-systolic and end-diastolic volumes, along with cardiac output metrics (EF, stroke volume (SV), cardiac output (CO), cardiac index (CI)) were obtained from all subjects (imaging and processing details are provided in Supplementary Material). Total scan time for the SSFP MRI was approximately 3 min.

#### Vascular Flow Imaging and Aortic Relative Pressure Estimation

**Fig. 2:**
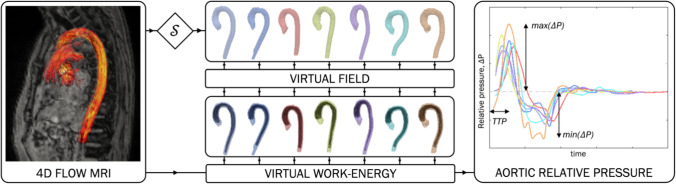
Overview of the virtual work-energy principle. Overview of the virtual work-energy principle to derive aortic relative pressure from 4D flow MRI (left). Aortic segmentations (*S*, upper mid-portion) are shown together with corresponding virtual fields (lower mid-portion), used to isolate aortic relative pressure. Maximum relative pressure, minimum relative pressure, and time-to-peak relative pressure (TTP) are derived (right). Each case is processed individually, with the colour coding of the segmentations corresponding to the ones of the relative pressure traces

All subjects were imaged using 4D flow MRI (eightfold acquisition using the k-t PCA technique [40] in combination with a sparsifying transform [27], spatial resolution ~ 2.5 mm^3^, temporal resolution ~ 33 ms, prospective ECG gating, velocity encoding range ~ 120–190 cm/s). Total scan time for the 4D flow MRI was approximately 9–17 min. The thoracic aorta was segmented using an in-house software, with aortic anatomical entities (mean curvature, mean length from aortic outflow to the diaphragm, mean diameter over the entire segmented section) quantified. Aortic relative pressure was computed from the left ventricular outflow tract (LVOT) to the diaphragm level of the descending aorta using a validated virtual work-energy approach (see Marlevi et al. [34] and Supplementary Material). From each relative pressure trace, maximum and minimum relative pressures were derived. Additionally, time-to-peak relative pressure (TTP) was computed, given as the time from acquisition onset (triggered at ECG R-wave) to maximum relative pressure. These three metrics were chosen to represent aortic hemodynamic behaviour, with positive and negative relative pressure relating to the acceleration and deceleration of blood through the aorta and with TTP relating to ventricular conduction [44] and myocardial contractility [42]. An illustration of the derived metrics is given in Fig. 2.

Aortic pulse wave velocity (PWV) was derived using a validated cross-correlation method, assessing the transit time between the prescribed inlet (at around the left ventricular outflow tract) and outlet (at around the diaphragmal level) flows and showing the lowest sensitivity to noise and low temporal resolution among reviewed methods [20]. Aortic stiffness was subsequently calculated using the Moens-Korteweg equation with subject-specific mean aortic radius and a constant wall thickness of 1.6 mm [37].

#### Central Blood Pressure Estimation

Central blood pressure (CBP) estimates were derived from brachial sphygmomanometer cuffing acquired prior to imaging, using dedicated equipment (CENTRON cBP301, SunTech Medical Inc., Morrisville, NC, USA) where brachial pressures are converted into CBP by means of validated transfer functions [8].

### Virtual Cohort

Structural and functional cardiovascular characteristics can be studied by means of non-invasive imaging. However, the interdependent nature of ventricular and vascular behaviour makes it difficult to isolate the effects of individual factors on cardiovascular function. Computational modelling, however, allows for the study of the physiological effects of specific parameters on the cardiovascular system in an isolated fashion [10]. To understand aortic relative pressures, a virtual cohort was thus created, solving 1D blood flow equations through a systemic circulatory model, all based on the models described in Willemet et al. [49] and Alastruey et al. [2]. Importantly, such models have been extensively validated and verified to accurately represent 1D arterial hemodynamic behaviour through the larger arterial system [38, 41].

The virtual cohort was adjusted to match clinical characteristics (arterial peripheral resistance, SV, cardiac period, aortic volumetric size, aortic stiffness), with both DCM_*red*_ and DCM_*pres*_ having a corresponding virtual subgroup. By varying isolated parameters around a subgroup baseline, the independent influence of these defined clinical characteristics was assessed. With one baseline set, and two permutations per isolated parameter, that created a virtual cohort size of 11 subjects. In all instances, virtual relative pressures were derived from the LVOT to the descending aorta, with outputs normalized over aortic length. Technical details of the virtual cohort are provided in Supplementary Material.

### Statistical Analysis

Statistical differences in subject characteristics, cardiac metrics, and aortic outputs were evaluated using a Mann–Whitney *U* test for continuous data and a χ^2^ test for nominal data (significance inferred at *p* < 0.05). Outliers were evaluated by Tukey’s fences. For continuous variables, 90% confidence intervals were also derived.

The Pearson correlation coefficient was evaluated to assess potential correlations between subject characteristics and output metrics. Correlation was inferred for |*R*|> 0.5 and *p* < 0.002 (determined from *p* < 0.05 together with a Bonferroni correction for *m* = 21 tested correlates, introduced to account for the multiple comparisons).

All evaluations were performed using MATLAB R2016a (MathWorks, Natick, MA, USA).

## Results

Upon evaluation, one DCM subject with reduced LV systolic function was excluded following the identification of mild aortic stenosis (maximum aortic outflow velocity = 1.8 m/s versus group mean = 0.8 ± 0.3 m/s, falling beyond the outer Tukey fence) and signs of altered aortic geometry (LVOT cross-sectional area = 1.5 cm^2^ versus a group mean = 5.9 ± 2.9 cm^2^). No signs of valvular disease were observed in any of the other subjects.

### Clinical Data and Subject Characteristics

Complete characteristics and data output are provided in Table 2. The DCM_*red*_ group showed significantly higher body mass index (BMI) and LV volumes and significantly lower LV EF, LV cardiac index (CI), as well as right ventricular (RV) CI compared to reference controls (with separation including full 90% confidence intervals). Systolic and mean blood pressures were also elevated. Beyond that, none of the derived anatomical measures on neither left nor the right heart indicated any statistical difference between groups. Note that none of the included patients had any previous incidences of either myocarditis or chemotherapy .

**Table 2:**
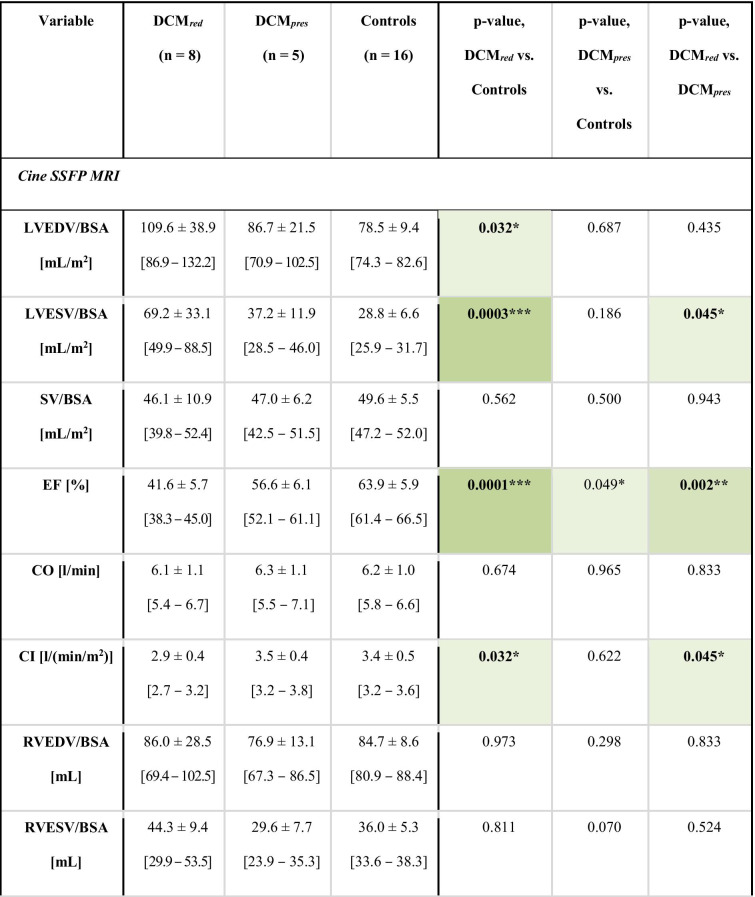

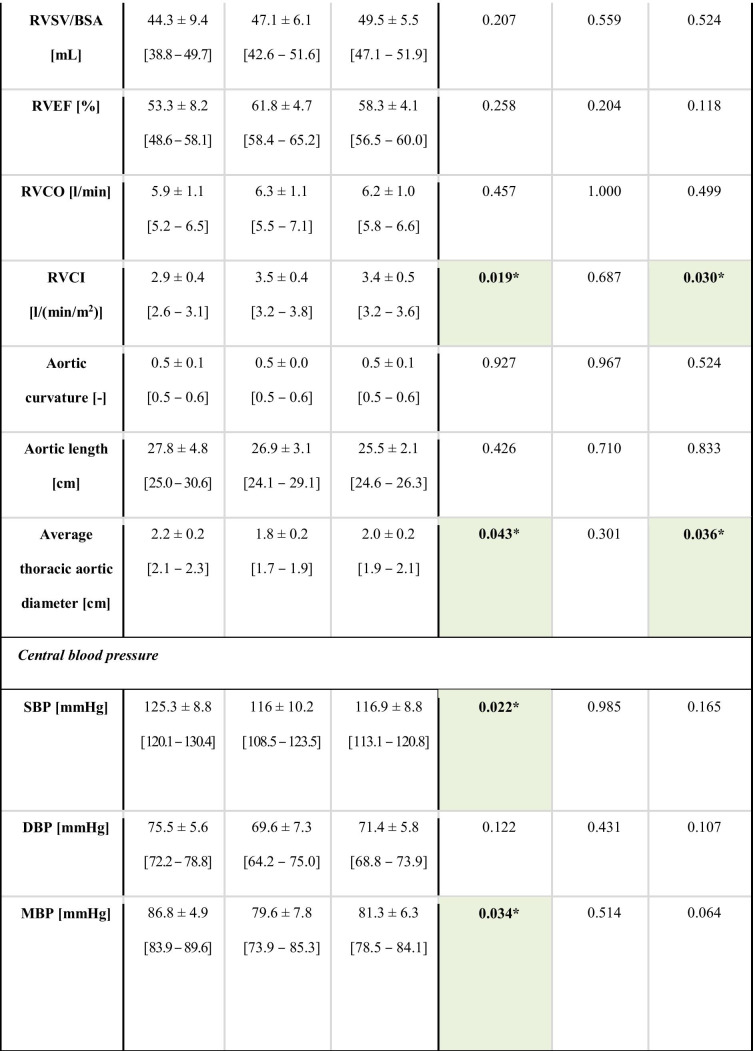
Data characteristics. Data and subject characteristics for the DCM_*red*_, DCM_*pres*_, and control groups, respectively. Intragroup *p*-values are reported with significant differences indicated by * (*p* < 0.05), ** (*p* < 0.01), or *** (*p* < 0.001), as well as with the colour coding. Volumes are normalized by body surface area (BSA). 90% confidence intervals are given in brackets.

**Table 3:**
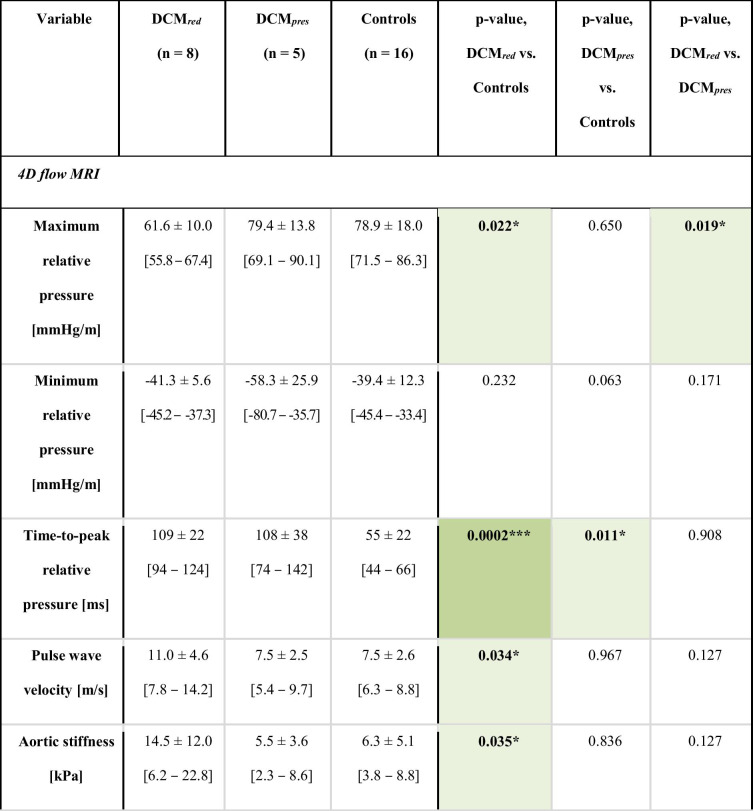
Aortic flow analysis. Aortic relative pressure metrics for the DCM_*red*_, DCM_*pres*_, and control groups, respectively. Intragroup *p*-values are reported with significant differences indicated by * (*p* < 0.05), ** (*p* < 0.01), or *** (*p* < 0.001), as well as with the colour coding. 90% confidence intervals are given in brackets.

### Aortic Relative Pressure in Dilated Cardiomyopathy Patients

**Fig. 3:**
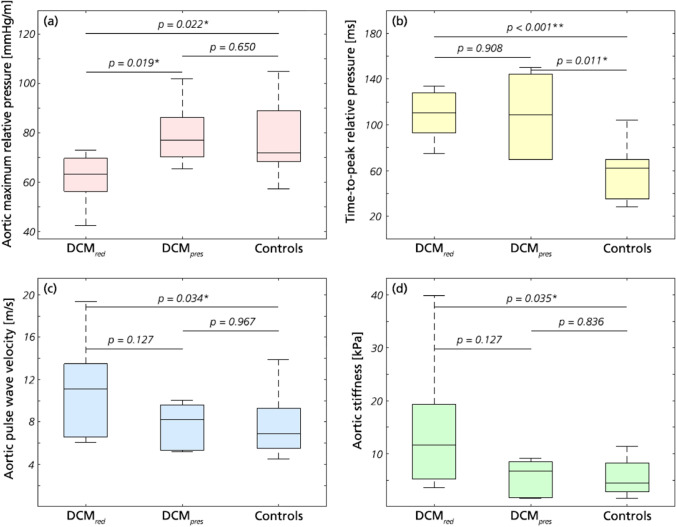
Key results from aortic relative pressure analysis. **a** Maximum relative pressure, **b** TTP, **c** PWV, and **d** aortic stiffness for DCM_*red*_, DCM_*pres*_, and the control groups, respectively. *p*-values are reported throughout with significance indicated by * (*p* < 0.05) or ** (*p* < 0.01)

Aortic relative pressure characteristics are provided in Table 3, with Fig. 3 showing comparisons of flow-derived markers.

The DCM_*red*_ group showed a significantly decreased maximum aortic relative pressure compared to both DCM_*pres*_ and reference controls, with an average reduction of 22% (61.6 ± 10.0 mmHg/m vs. 78.9 ± 18.0 mmHg/m, p = 0.022). The differentiation also included complete separation of 90% confidence intervals. However, this difference was not reflected for the minimum relative pressure, where similar values were obtained as for the control group.

A markedly prolonged TTP was seen in both DCM_*red*_ and DCM_*pres*_ groups compared to reference controls. Strong statistical differences in group means were seen between the control group and both DCM groups (*p* ≤ 0.01), with the DCM patients requiring twice as much time to reach maximum relative pressure (109 ± 22 and 108 ± 38 ms vs. 55 ± 22 ms). Again, inferences included complete separation of 90% confidence intervals.

The DCM_*red*_ group showed a higher aortic PWV compared to the reference control group, with an increase of 47% (11.0 ± 4.6 m/s vs. 7.5 ± 2.5 m/s, *p* = 0.034). Increased PWV was not noted in the DCM_*pres*_. Similar results were observed for derived aortic stiffness, where the DCM_*red*_ group had a significantly higher aortic stiffness compared to reference controls, with an increase of 130% (14.5 ± 12.0 kPa vs 6.3 ± 5.1 kPa, *p* = 0.035). The separation was not strong enough to completely separate the 90% confidence intervals of respective groups.

### Correlation of Aortic Relative Pressure with Clinical, Structural, and Central Blood Pressure Metrics

Complete correlation results are provided in Supplementary Table 2. In short, for the DCM_*red*_ and DCM_*pres*_ groups, no correlations could be inferred between any clinical parameter and the derived aortic metrics, including central blood pressures. For the control group, similar behaviour was observed, with only BSA and BMI correlated to minimum relative pressure and aortic stiffness, respectively. Noteworthy, the absence of correlation spanned both left and right heart metrics, highlighting the independent role of our derived aortic variables.

### Influence of Isolated Cardiovascular Properties on Aortic Relative Pressure

**Fig. 4:**
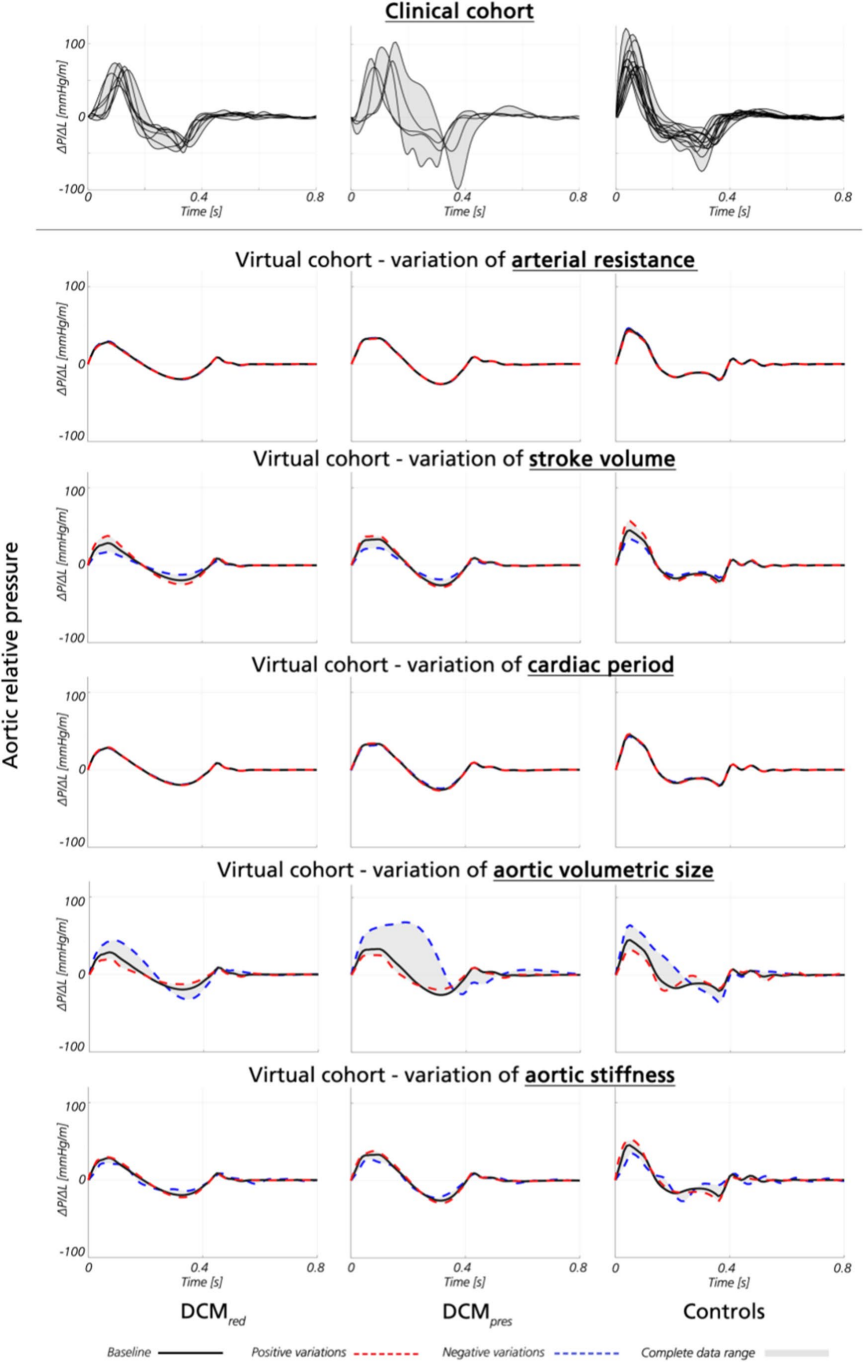
Aortic relative pressure traces from clinical and virtual analysis. Aortic relative pressure traces from the clinical (top row) and virtual cohort (remaining rows), presented as a function of isolated variations of arterial resistance, stroke volume, cardiac period, aortic volumetric size, and aortic stiffness, respectively. Relative pressure is shown for DCM_*red*_ (left), DCM_*pres*_ (middle), and controls (right). For the clinical data, each individual subject is given in black. For the virtual data, isolated variations around baseline (black) are given as positive variations (red) and negative variations (blue), respectively, superimposed on the data range (grey)

The individual influence of cardiovascular properties on aortic relative pressure was analysed using the virtual cohort. Aortic relative pressure for each virtual subgroup, together with corresponding clinical traces, is shown in Fig. 4.

For the virtual cohort, aortic maximum relative pressure varied between 17.2–44.3, 22.1–68.0, and 32.6–64.3 mmHg/m for the DCM_*red*_, DCM_*pres*_, and control groups, respectively. For reference, the clinical cohort varied between 42.4–72.8, 62.2–102.7, and 57.3–121.4 mmHg/m within the same three groups, respectively. Individual variations as a function of isolated variables are given in Supplementary Table 3.

Aortic stiffness and aortic volumetric size (the total volume of the segmented aorta) were the two dominant properties influencing aortic relative pressure in the virtual cohort. Specifically, variations in aortic stiffness alone recovered 28% of the total variations observed in maximum relative pressure in the DCM_*red*_ subgroup, 23% in the DCM_*pres*_ group, and 56% in the control group. Similarly, variations in aortic volumetric size alone recovered 89, 92, and 100% of the total observed variations in maximum relative pressure in the same three groups, respectively. In contrast, SV recovered 76, 34, and 50% of the total observed variations, whilst cardiac period and peripheral resistance had a comparatively smaller influence (both influencing relative pressures < 2 mmHg/m across all sets).

For minimum relative pressure, similar behaviour could be observed where aortic stiffness and aortic volumetric size were the two major contributors to the observed variations, with aortic stiffness equalling 40, 64, and 5% of the total variations and with aortic volumetric size equalling 98, 53, and 78%, again reported for the DCM_*red*_, DCM_*pres*_, and control groups, respectively. Individual variations as a function of isolated variables are given in Supplementary Table 4.

## Discussion

In this study, we have used 4D flow MRI to explore aortic hemodynamic changes in patients with idiopathic DCM. We identified differences in aortic function in DCM patients with reduced LV systolic function as compared to those with preserved systolic function or healthy volunteers, with decreasing maximum aortic relative pressure and increased aortic stiffness indicated by the flow-based analysis. Possible signs of ventricular conduction delay were also provided by the aortic analysis, with prolonged TTP evident in DCM patients, regardless of LV function. Complementing these clinical observations, our computational virtual cohort showed how aortic hemodynamic metrics are governed in part by arterial properties (aortic size and stiffness), suggesting—in conjunction with our clinical findings—the potential role of *vascular* adaptation in the pathological manifestation of a *cardiac* disease (i.e. DCM). Given previous observations linking cardiac disease and treatment effect to arterial behaviour [36, 51], and the fact that recent consensus documents highlight the role of ventricular-vascular interactions in heart failure [23], our exploratory study exemplifies how analysis of vascular function using CMR could complement the clinical assessment of DCM patients and how such a combined assessments of cardiac and arterial function may yield new insights and novel hypotheses.

### Changes in Aortic Relative Pressure—Relation to Cardiac and Aortic Physiology

The DCM_*red*_ group showed a significantly reduced maximum aortic relative pressure, being on average 22% lower than the control group. Conversely, the DCM_*pres*_ group did not differ significantly from the control, maintaining maximum aortic relative pressure. Whilst the DCM_*red*_ and DCM_*pres*_ groups are defined on the basis of ventricular function, the observed differences in aortic relative pressure suggests how changes in cardiac and vascular function are interlinked in patients with DCM. In previous studies, DCM has been associated with a reduction in peak aortic outflow acceleration [24], and deteriorating cardiovascular status has been correlated to a decrease in LVOT outflow gradients [3], similarly highlighting a link between ventricular dilation and affected vascular hemodynamics. Given that aortic relative pressure has been correlated to LV remodelling [7], our findings suggest that vascular alterations are present in idiopathic DCM. Additionally, the observed differences between DCM_*red*_ and DCM_*pres*_ suggest a possible role of vascular assessment in providing a more nuanced assessment of disease status and disease progression.

In contrast, no significant differences could be inferred for minimum aortic relative pressure, where both DCM_*red*_ and DCM_*pres*_ groups showed similar values to the control group. Minimum aortic relative pressure—an entity related to the deceleration of blood during late systole—has been less studied in conjunction with cardiac disease but has for aortopathies (e.g. bicuspid aortic valve or aortic dissection) been related to a composite of cardiac and aortic physiology [28]. Whether this holds true for the link between aortic relative pressure and idiopathic DCM requires further evaluation.

In comparison to the above outlined links between maximum relative pressure and aortic function, TTP is an entity directly coupled to cardiac contractility: the shorter the TTP, the faster the ventricular contraction. In our study, a significant delay in TTP was observed in both DCM groups relative to controls, with an almost doubling of the time required to reach maximum relative pressure. Ventricular conduction delay is commonly reported for DCM [26], and although conduction abnormalities were not directly assessed in our study, prolonged QRS duration has been correlated to a reduction in maximum intraventricular relative pressure [50], in agreement with our findings with aorta-derived TTP. Noteworthy is the fact that both DCM groups exhibited prolonged TTP, regardless of LV functional status. That is, even though LV systolic function was decreased in some and preserved in others, the consistently prolonged TTP indicates underlying electrophysiological abnormalities in both groups. In DCM, ventricular conduction delay has shown to be one of the most powerful predictors of prognosis [46], and with idiopathic DCM connected to long-term development of heart failure [18], latent contractile abnormalities—as suggested by the prolonged TTP in our cohort—could have detrimental long-term ramifications even in groups where an apparent improvement in cardiac ejection fraction is observed.

A significant increase in PWV and aortic stiffness estimates were obtained in the DCM_*red*_ group, with aortic stiffness being almost 2.3 times higher as compared to the reference control group (although without complete separation of 90% confidence intervals). On the contrary, the DCM_*pres*_ group did not show the same stiffness increase. Arterial stiffening in DCM patients has been described previously [5], and reduced maximum relative pressure was also recently reported in an elderly cohort [7]. Worth noting is also that guideline recommended treatment for heart failure includes administration of vasodilators, diuretics, and beta blockers: medications all shown to *decrease* PWV and vascular stiffness [31, 39]. The fact that our data indicates opposite behaviour highlights the potential presence of other mechanisms overruling the intended therapeutic outcome.

Lastly, the DCM_*red*_ group showed significantly increased systolic blood pressure compared to the control group. This increase in afterload is however not coupled to any significant differences in SV, suggesting that increased ejection force is required to maintain systemic circulation in the DCM_*red*_ group. Furthermore, the simultaneous increase in mean blood pressure indicates elevated systemic resistance in the DCM_*red*_ group; however, this is slightly opposed by unaltered diastolic blood pressure and maintained cardiac output. In general, the development of hypertension is common in DCM [13] and originates in part from increased neurohormonal activation [9]. It highlights a persistent detrimental feedback loop, in which long-term cardiac congestion leads to increased afterload due to sympathetic activation. The chronic overload of the ventricle thus in turn leads to worsening contractile properties [9]. This form of ventricular-vascular coupling in the presence of hypertension has also been highlighted in summarized review work [43], detailing how a range of hypertension-related arterial changes propagate into cardiac behaviour and long-term function. Pinpointing specific mechanisms of relevance in relation to our observed results, hypertension-related aortic stiffening renders an early aortic pressure pulse return. Importantly, this not only elevates systolic afterload and increases required LV ejection force, but *also* decreases diastolic coronary perfusion and coupled myocardial fibre relaxation, together exemplifying how modified aortic hemodynamics can have a profound impact on cardiac behaviour throughout the cardiac cycle (more on the clinical implications of our findings in subsequent subsections).

### Correlation to Aortic Relative Pressure—Independent Role of Aortic Hemodynamics

The correlation analysis revealed a general lack of defined relationships between aortic hemodynamics and standard volumetric and functional metrics for the entire DCM cohort, including both left and right heart metrics. Even though this might be an artefact from the limited sample size, these results should still be contrasted to the clear differences in aortic metrics between DCM_*red*_ and DCM_*pres*_ groups. Further investigation is required to establish the case of these differences; however, the differences in aortic relative pressure between DCM subgroups—even in a limited cohort—highlights how aortic assessment may complement standard cardiac metrics in DCM patients. Others have indicated the independent association between aortic relative pressure and LV remodelling [7], specifically highlighting relative pressure as a potential complementary and independent biomarker in refined cardiac diagnosis. Lastly, although no indications of correlation were observed between right heart metrics and aortic hemodynamics, the scarcity of such an assessment in the literature could warrant further evaluation in this specific direction.

### The Role of Cardiac and Aortic Function on Relative Pressure—Isolated Virtual Cohort Study

Out of the evaluated metrics in the virtual cohort analysis, aortic stiffness and mean cross-sectional volume were the main determinants of aortic relative pressure. However, whilst *positive* changes in stiffness *increase* relative pressure magnitudes, positive changes in volume *decrease* them. Such opposing effects could explain the maintained minimum relative pressure in the DCM group however would not fully clarify the corresponding net decrease in maximum relative pressure. This reduction in maximum relative pressure could instead be attributed to cardiac changes such as modified contractility [22], lack of contractile coordination [26], or vasodilating medication both increasing aortic dimensions and attempting to reduce effective vascular stiffness [31, 39]; however, clarification of such requires further analysis in extended cohorts or using alternative computational assessments using, e.g. higher-order 3D modelling. Regardless, our combination of clinical and virtual cohorts supports the hypothesis that—in the presence of idiopathic DCM—abnormalities of cardiac *and* vascular properties co-exist.

### Aortic Change in DCM—Clinical Implications of Image-Based Findings

In our exploratory study, a number of aortic hemodynamic differences have been indicated for idiopathic DCM, with specific differences highlighted between DCM_*red*_ and DCM_*pres*_. Importantly, with the two DCM groups undergoing similar guideline-based medical therapy, the separation could represent an initial indicator to how cardiac treatment efficacy may be, at least in part, coupled to vascular function.

Antihypertensive medications have been shown to reduce arterial stiffness [15], being part of a general strategy to hinder continuous ventricular dilation through cardiac unloading. However, in the presence of pathological vascular changes—such as elevated arterial stiffness in the DCM_*red*_ group—an ineffective treatment response can be hypothesized: the composite scenario of increased blood pressure *and* aortic stiffness could increase the amount of myocardial work required to maintain systemic perfusion. Furthermore, with increasing degrees of aortic dilation leading to decreased relative pressure, systemic perfusion may be negatively effected. If, at this stage, additional pharmacological interventions cause decreased aortic stiffness or blood pressure, further unfavourable reductions in relative pressure could follow. Along the same lines, alternative but complementary mechanisms could also be present: whilst healthy arteries see the aortic pressure pulse reflected during late diastole, in diseased stiff arteries, the reflected pressure wave arrives already during systole [43]. If so, an increase in systolic blood pressure and LV afterload will be observed, decreasing both aortic relative pressures and prolonging effective ejection time—again, in line with our findings. The latter will also result in detrimental LV remodelling including increased oxygen demand following changes in afterload, decreased coronary perfusion resulting from an early aortic pressure return, and increased LV diastolic filling pressures arising from the prolonged ejection time. Whilst the causal relationships between these factors remains to be determined in dedicated studies with expanded cohorts, the above reasoning exemplifies how poor vascular function could in fact influence the ability to achieve optimal cardiac unloading and how such arterial behaviour is important to consider in the therapeutic management of cardiac patients. Whilst using arterial function as a measure of treatment efficacy is not a novel concept [12], our study highlights that unloading the heart requires a unified look at cardiac, aortic, and systemic vascular function.

### Clinical Outlook—Non-invasive Imaging for the Assessment of Ventricular-Vascular Function

Blood flow assessment by advanced MRI permits detailed hemodynamic analysis. As shown in our exploratory study, aortic hemodynamics appear altered in cardiac disease, and aortic relative pressure may in fact provide insights into the summation of cardiac and vascular function, complementing what can be inferred by conventional cardiac assessment alone. With physics-based image processing allowing for the pressure metrics to be derived directly from non-invasively acquired data [32–35], such metrics could serve as important biomarkers, especially in diseases where hemodynamic assessment has traditionally relied on invasive catheterization. Several examples exist of where volumetric flow imaging has been suggested to improve the assessment of cardiovascular disease: quantifying 4D intraventricular flow in relation to heart failure severity [45] or assessing aortic hemodynamics in conjunction to ventricular remodelling [7]. Our study brings another potential clinical application of advanced flow imaging in assessing arterial alterations in idiopathic DCM. Longitudinal studies are required to uncover the initiation and ordering of physiologic responses; however, imaging has the potential to play a key role in studying such behaviour in a non-invasive setting. With accelerated sequence protocols promising more rapid acquisition times [4, 25], and considering our virtual cohort indicating aortic hemodynamic as invariant to cardiac period, our study also highlights the feasibility of using 4D flow MRI in a direct clinical setting.

### Limitations

A fairly small sample size was used, and the generalization of these findings to a global DCM population requires further investigation. Likewise, being a retrospective study, we were not able to control for differences in unmeasured patient characteristics and treatments between groups, although patients all followed guideline-directed therapy. In particular, this effect is enhanced by the post-hoc splitting of the patient cohort into DCM_*red*_ and DCM_*pres*_, effectively reducing subgroup sample sizes from which to derive statistical inferences. To provide estimates of reliability, confidence intervals are reported, highlighting how separation with respect to aortic relative pressures and TTP seem corroborated even in our comparably small cohort (with separation strong enough to avoid overlapping confidence intervals), whereas separation with respect to PWV and aortic stiffness bears less statistical confidence (with confidence intervals overlapping between evaluated groups). Although to be used with caution [30, 47, 52], post-hoc power analysis also indicates similar predictive abilities, where the power in differentiating DCM_*red*_ vs. controls with respect to relative pressures or TTP is consistently above 1-β = 0.85 (at *α* = 0.05), whilst estimates of PWV and aortic stiffness comes with 1-β around 0.50 (at *α* = 0.05). Keeping the above in mind, we therefore view our results not as definite evidence of specific mechanisms but rather as an indicative example of how advanced flow imaging together with biophysical modelling can provide hypothesis-generating insights into pathophysiological developments.

For the virtual cohort, the analysis was limited to a selection of key cardiovascular properties, chosen based on their conventional use in cardiovascular practice. The virtual cohort showed slightly lower relative pressure compared to the retrieved clinical data in all three subgroups. As is common in 1D cardiovascular simulations, systemic baseline characteristics (peripheral resistance, systemic variations in PWV, segment diameters, etc.) were retrieved from published literature values on healthy subjects, with only conventionally assessable metrics (SV, heart rate) tailored to reflect certain pathologies. Consequently, these underlying baseline characteristics might not be entirely reflective of the assessed clinical cohort and might cause the observed bias. However, with virtual and clinical cohorts showing similar trends with respect to aortic relative pressure (decreasing relative pressure with increasing LV impairment), there are reasons to believe that the isolated influence of evaluated cardiovascular parameters on the virtual cohort is reflective of similar behaviour on the clinical side.

Minor technical limitations might also exist in the acquired data, with the lack of arrhythmia correction possibly causing unwanted variations in the presence of ectopic heart beats or irregular cardiac rhythms. However, with data acquired over several cardiac cycles, and with the virtual cohort analysis revealing how aortic metrics are comparably stable under varying heart rates, the impact of such potential variations should be considered minor and not to have overly affected our derived results.

## Conclusion

In this study, a combination of non-invasive MRI blood flow imaging and computational modelling were used to study aortic relative pressure in DCM. Significant differences in aortic hemodynamics were observed between DCM patients and healthy volunteers, as well as within DCM subgroups, with key observations including decreased maximum relative pressure coupled to impaired ventricular ejection (DCM_*red*_ group), prolonged TTP suggesting the presence of conduction delay (both DCM groups regardless of LV status), and increased aortic stiffness and systolic blood pressure suggesting concurrent arterial remodelling (DCM_*red*_ group). Whilst the causal relationships needs to be further investigated in larger longitudinal cohorts and experimental studies, given that the aortic properties were different between patients with preserved vs. reduced LV systolic function, we believe that vascular adaptation—as assessed by 4D flow-derived aortic stiffness and relative pressure—could complement the existing techniques for assessing disease severity and treatment effects in DCM. Lastly, this study highlights the advantages of combined 4D flow and biophysical image processing in the non-invasive assessment of ventricular-vascular abnormalities in vivo.

## Supplementary Information

Below is the link to the electronic supplementary material.

Supplementary file1 (DOCX 278 KB)

## References

[1] Abramson SV, Burke JF, Kelly JJ (1992). Pulmonary hypertension predicts mortality and morbidity in patients with dilated cardiomyopathy. Annals of Internal Medicine.

[2] Alastruey J, Parker KH, Sherwin SJ (2012). Arterial pulse wave haemodynamics. 11th International Conference on Pressure Surges.

[3] Avramides D, Perakis A, Voudris V (2000). Noninvasive assessment of left ventricular systolic function by stress-shortening relation, rate of change of power, preload-adjusted maximal power, and ejection force in idiopathic dilated cardiomyopathy: Prognostic implications. Journal of the American Society of Echocardiography.

[4] Bollache E, Barker AJ, Dolan RS (2018). k-t accelerated aortic 4D flow MRI in under two minutes: Feasibility and impact of resolution, k-space sampling patterns, and respiratory navigator gating on hemodynamic measurements. Magnetic Resonance in Medicine.

[5] Bonapace S, Rossi A, Cicoira M et al. (2006) Aortic stiffness correlates with an increased extracellular matrix turnover in patients with dilated cardiomyopathy. Am. Heart J. 152:93. e91–93. e96 10.1016/j.ahj.2006.04.026 16824836

[6] Borlaug BA, Kass DA (2011). Ventricular–vascular interaction in heart failure. Cardiology Clinics.

[7] Bouaou K, Bargiotas I, Dietenbeck T (2019). Analysis of aortic pressure fields from 4D flow MRI in healthy volunteers: Associations with age and left ventricular remodeling. Journal of Magnetic Resonance Imaging.

[8] Brett SE, Guilcher A, Clapp B (2012). Estimating central systolic blood pressure during oscillometric determination of blood pressure: Proof of concept and validation by comparison with intra-aortic pressure recording and arterial tonometry. Blood Pressure Monitoring.

[9] Bristow MR (1984) The adrenergic nervous system in heart failure. In:Mass Medical Soc 10.1056/NEJM198409273111310 6472388

[10] Chabiniok R, Wang VY, Hadjicharalambous M (2016). Multiphysics and multiscale modelling, data–model fusion and integration of organ physiology in the clinic: Ventricular cardiac mechanics. Interface Focus.

[11] Chirinos JA, Sweitzer N (2017). Ventricular-arterial coupling in chronic heart failure. Cardiac Failure Review.

[12] Cohn JN, Duprez DA, Grandits GA (2005). Arterial elasticity as part of a comprehensive assessment of cardiovascular risk and drug treatment. Hypertension.

[13] Dec GW, Fuster V (1994). Idiopathic dilated cardiomyopathy. New England Journal of Medicine.

[14] Donati F, Myerson S, Bissell MM et al. (2017) Beyond Bernoulli. Circ. Cardiovasc. Imaging 10:e005207 10.1161/CIRCIMAGING.116.005207 PMC5265685 28093412

[15] Dudenbostel T, Glasser SP (2012). Effects of antihypertensive drugs on arterial stiffness. Cardiology in Review.

[16] Dyverfeldt P, Bissell M, Barker AJ (2015). 4D flow cardiovascular magnetic resonance consensus statement. Journal of Cardiovascular Magnetic Resonance.

[17] Eriksson J, Bolger AF, Ebbers T (2013). Four-dimensional blood flow-specific markers of LV dysfunction in dilated cardiomyopathy. European Heart Journal-Cardiovascular Imaging.

[18] Felker GM, Thompson RE, Hare JM (2000). Underlying causes and long-term survival in patients with initially unexplained cardiomyopathy. New England Journal of Medicine.

[19] Ferreira VM, Piechnik SK, Robson MD (2014). Myocardial tissue characterization by magnetic resonance imaging: Novel applications of T1 and T2 mapping. Journal of Thoracic Imaging.

[20] Gaddum N, Alastruey J, Beerbaum P (2013). A technical assessment of pulse wave velocity algorithms applied to non-invasive arterial waveforms. Annals of Biomedical Engineering.

[21] Gersh BJ, Maron BJ, Bonow RO (2011). 2011 ACCF/AHA guideline for the diagnosis and treatment of hypertrophic cardiomyopathy: A report of the American College of Cardiology Foundation/American Heart Association Task Force on practice guidelines. Journal of the American College of Cardiology.

[22] Hasenfuss G, Mulieri L, Leavitt B (1992). Alteration of contractile function and excitation-contraction coupling in dilated cardiomyopathy. Circulation Research.

[23] Ikonomidis I, Aboyans V, Blacher J (2019). The role of ventricular–arterial coupling in cardiac disease and heart failure: Assessment, clinical implications and therapeutic interventions. A consensus document of the European Society of Cardiology Working Group on Aorta & Peripheral Vascular Diseases, European Association of Cardiovascular Imaging, and Heart Failure Association. European Journal of Heart Failure.

[24] Isaaz K, Pasipoularides A (1991). Noninvasive assessment of intrinsic ventricular load dynamics in dilated cardiomyopathy. Journal of the American College of Cardiology.

[25] Jung B, Stalder AF, Bauer S (2011). On the undersampling strategies to accelerate time-resolved 3D imaging using k-t-GRAPPA. Magnetic Resonance in Medicine.

[26] Kerwin WF, Botvinick EH, O’connell JW (2000). Ventricular contraction abnormalities in dilated cardiomyopathy: Effect of biventricular pacing to correct interventricular dyssynchrony. Journal of the American College of Cardiology.

[27] Knobloch V, Boesiger P, Kozerke S (2013). Sparsity transform k-t principal component analysis for accelerating cine three-dimensional flow measurements. Magnetic Resonance in Medicine.

[28] Lamata P, Pitcher A, Krittian S (2014). Aortic relative pressure components derived from four-dimensional flow cardiovascular magnetic resonance. Magnetic Resonance in Medicine.

[29] Lee N, Taylor MD, Banerjee RK (2015). Right ventricle-pulmonary circulation dysfunction: A review of energy-based approach. Biomedical engineering online.

[30] Levine M, Ensom MH (2001) Post hoc power analysis: An idea whose time has passed? Pharmacotherapy: The Journal of Human Pharmacology and Drug Therapy 21:405–409 10.1592/phco.21.5.405.34503 11310512

[31] Mallareddy M, Parikh CR, Peixoto AJ (2006). Effect of angiotensin-converting enzyme inhibitors on arterial stiffness in hypertension: Systematic review and meta-analysis. The Journal of Clinical Hypertension.

[32] Marlevi D, Balmus M, Hessenthaler A (2021). Non-invasive estimation of relative pressure for intracardiac flows using virtual work-energy. Med. Image Anal..

[33] Marlevi D, Ha H, Dillon-Murphy D (2020). Non-invasive estimation of relative pressure in turbulent flow using virtual work-energy. Med. Image Anal..

[34] Marlevi D, Ruijsink B, Balmus M (2019). Estimation of cardiovascular relative pressure using virtual work-energy. Science and Reports.

[35] Marlevi D, Schollenberger J, Aristova M et al. (2021) Non-invasive quantification of cerebrovascular pressure changes using 4D flow MRI. in-press 10.1002/mrm.28928 PMC11421438 34431550

[36] Merlo M, Caiffa T, Gobbo M (2018). Reverse remodeling in dilated cardiomyopathy: Insights and future perspectives. IJC heart & vasculature.

[37] O'rourke MF (2007). Arterial aging: Pathophysiological principles. Vascular Medicine.

[38] Olufsen MS, Peskin CS, Kim WY (2000). Numerical simulation and experimental validation of blood flow in arteries with structured-tree outflow conditions. Annals of Biomedical Engineering.

[39] Ong K-T, Delerme S, Pannier B (2011). Aortic stiffness is reduced beyond blood pressure lowering by short-term and long-term antihypertensive treatment: A meta-analysis of individual data in 294 patients. Journal of Hypertension.

[40] Pedersen H, Kozerke S, Ringgaard S (2009). k-t PCA: Temporally constrained k-t BLAST reconstruction using principal component analysis. Magnetic Resonance in Medicine: An Official Journal of the International Society for Magnetic Resonance in Medicine.

[41] Reymond P, Bohraus Y, Perren F (2011). Validation of a patient-specific one-dimensional model of the systemic arterial tree. American Journal of Physiology-Heart and Circulatory Physiology.

[42] Ruijsink B, Zugaj K, Wong J (2020). Dobutamine stress testing in patients with Fontan circulation augmented by biomechanical modeling. PLoS One.

[43] Saba PS, Cameli M, Casalnuovo G (2014). Ventricular–vascular coupling in hypertension: Methodological considerations and clinical implications. Journal of Cardiovascular Medicine.

[44] Sermesant M, Chabiniok R, Chinchapatnam P (2012). Patient-specific electromechanical models of the heart for the prediction of pacing acute effects in CRT: A preliminary clinical validation. Medical Image Analysis.

[45] Stoll VM, Hess AT, Rodgers CT (2019). Left ventricular flow analysis: Novel imaging biomarkers and predictors of exercise capacity in heart failure. Circ. Cardiovasc. Imaging.

[46] Unverferth DV, Magorien RD, Moeschberger ML (1984). Factors influencing the one-year mortality of dilated cardiomyopathy. The American journal of cardiology.

[47] Walters SJ (2009). Consultants’ forum: Should post hoc sample size calculations be done?. Pharmaceutical Statistics: The Journal of Applied Statistics in the Pharmaceutical Industry.

[48] Weber T, Auer J, Lamm G (2007). Arterial stiffness, central blood pressures, and wave reflections in cardiomyopathy—Implications for risk stratification. Journal of Cardiac Failure.

[49] Willemet M, Chowienczyk P, Alastruey J (2015). A database of virtual healthy subjects to assess the accuracy of foot-to-foot pulse wave velocities for estimation of aortic stiffness. American Journal of Physiology-Heart and Circulatory Physiology.

[50] Xiao HB, Brecker SJ, Gibson DG (1992). Effects of abnormal activation on the time course of the left ventricular pressure pulse in dilated cardiomyopathy. Heart.

[51] Zanon F, Aggio S, Baracca E (2008). Ventricular-arterial coupling in patients with heart failure treated with cardiac resynchronization therapy: May we predict the long-term clinical response?. European Journal of Echocardiography.

[52] Zhang Y, Hedo R, Rivera A (2019). Post hoc power analysis: Is it an informative and meaningful analysis?. Gen Psychiatr.

