# Supplementary material for: Altered Aortic Hemodynamics and Relative Pressure in Patients with Dilated Cardiomyopathy
Source: J Cardiovasc Transl Res. 2021 Dec 9;15(4):692–707. doi: 10.1007/s12265-021-10181-1 (PMC9622552; doi:10.1007/s12265-021-10181-1)
Supplement: Supplementary file 1 — Supplementary file1 (DOCX 278 KB) [file 12265_2021_10181_MOESM1_ESM.docx]

**Supplementary material**

**­Altered aortic hemodynamics and relative pressure in patients with dilated cardiomyopathy**

David Marlevi^1,2,3,*^ PhD, Jorge Mariscal-Harana^4,*^ PhD, Nicholas S Burris^5^ MD,
Julio Sotelo^6,7,8^ PhD, Bram Ruijsink^4^ MD PhD, Myrianthi Hadjicharalambous^4,9^ PhD,
Liya Asner^4^ PhD, Eva Sammut^4,10^ MD PhD, Radomir Chabiniok^4,11,12,13^ MD PhD,
Sergio Uribe^7,8,14^ PhD, Reidar Winter^3^ MD PhD, Pablo Lamata^4^ PhD, Jordi Alastruey^4,15^ PhD,
David Nordsletten^4,16,+^ PhD

*****Shared first authorship

**Affiliations**

^1^ Institute for Medical Engineering and Science, Massachusetts Institute of Technology, MA, USA
^2^ Department of Biomedical Engineering and Health Systems, KTH Royal Institute of Technology, Huddinge, Sweden
^3^Department of Clinical Sciences, Karolinska Institutet, Danderyd, Sweden
^4^School of Biomedical Engineering and Imaging Sciences, King’s College London, London, United Kingdom
^5^ Department of Radiology, University of Michigan, Ann Arbor, MI, USA
^6^School of Biomedical Engineering, Universidad de Valparaíso, Valparaíso, Chile
^7^ Biomedical Imaging Center, Pontificia Universidad Catolica de Chile, Santiago, Chile
^8^ Millennium Nucleus in Cardiovascular Magnetic Resonance, Cardio MR, Santiago, Chile ^9^ Department of Mechanical and Manufacturing Engineering, University of Cyprus, Nicosia, Cyprus
^10^ Bristol Heart Institute and Translational Biomedical Research Centre, Faculty of Health Science, University of Bristol, Bristol, United Kingdom
^11^ Inria, Palaiseau, France
^12^ LMS, Ecole Polytechnique, CNRS, Institut Polytechnique de Paris, Paris, France
^13^ Department of Mathematics, Faculty of Nuclear Sciences and Physical Engineering, Czech Technical University in Prague, Prague, Czech Republic ^14^ Department of Radiology, School of Medicine, Pontifica Universidad Católica de Chile, Santiago, Chile
^15^ World-Class Research Center “Digital Biodesign and Personlized Healthcare”, Sechenov University, Moscow, Russia
^16^ Department of Surgery and Biomedical Engineering, University of Michigan, USA

**Corresponding author** (**+**)

| David Nordsletten  Dept. Cardiac Surgery and Biomedical Engineering, University of Michigan  Plymouth Rd, 48109  Ann Arbor, MI, USA  E-mail: [david.nordsletten@gmail.com](mailto:david.nordsletten@gmail.com)  Tel: +1 734 647 7000 Fax: +1 734 936 1905 |
| --- |

***Supplementary methods***

Structural magnetic resonance protocol and analysis

For structural analysis, cine SSFP MRI (retrospective ECG gating, spatial resolution ~2x2x8 mm^3^, temporal resolution ~20 ms) and 3D tagged MRI (prospective ECG triggering, spatial resolution ~3.4x3.4x7.7 mm^3^, temporal resolution ~30 ms) was acquired, all using a 1.5T Philips ACHIEVA system (Philips Healthcare Best, The Netherlands).

Left and right ventricular volumes (end-diastolic volumes (LVEDV, RVEDV) end-systolic volumes (LVESV, RVESV)), as well as left and right ventricular output metrics such as ejection fraction, stroke volume and cardiac output (EF, SV, CO, RVEF, RVSV, RVCO) were calculated by manually identifying end-diastolic and end-systolic segmentations of the short-axis images (CVI software, Circle Cardiovascular Imaging Inc, Alberta, Canada).

Mathematical details of the derivation of relative pressure from 4D flow MRI

From the 4D flow MRI data, relative pressure was derived using a virtual work-energy approach – $\nu$WERP – originally presented and validated against invasive catheter measurements in Marlevi et al^15^. The method originates from the Navier-Stokes equation, in which the conservation of mass and momentum are used to constrain the 3D movement of Newtonian fluid over time. From such, $\nu$WERP estimates the relative pressure $\Delta p$ across an arbitrary vascular section $\Omega$ with inlet plane $\Gamma_{i}$ and outlet plane $\Gamma_{o}$ as

| $\Delta p= -\frac{1}{Q}\left( \frac{\partial}{\partial t}K_{e}+A_{e}+V_{e} \right).$ | (A) |
| --- | --- |

Each entry in equation (A) now represents separated *virtual* energy components of an introduced *virtual* field, created in conjunction to the acquired 4D flow MRI. Specifically $Q$ is the virtual flow through the inlet, $K_{e}$ the virtual kinetic energy held within the fluid, $A_{e}$ the rate at which virtual kinetic energy changes within the field, and $V_{e}$ the rate of virtual viscous energy dissipation by the field.

With the above, $\nu$WERP acts directly on the non-invasively acquired 4D flow MRI data, and does not require any additional measurements to compute $\Delta p$. Specific details on data post-processing of the acquired velocity field, $\boldsymbol{v}$**,** as well as the creation of the auxiliary virtual field, $\boldsymbol{w}$, are all described using an identical setup in Marlevi et al^15^.

Virtual cohort creation and analysis

A virtual cohort was utilized based on Willemet et al^18^, where blood pressure, blood flow, and arterial area waveforms could be simulated using a validated one-dimensional (1D) computational model of arterial hemodynamics consisting of the 55 larger arteries of the human systemic circulation^27^. The virtual cohort was adapted to match the clinical characteristics, creating three virtual subgroups: one virtual DCM*_red_* group, one virtual DCM*_pres_* group, and one virtual control group, respectively. Specific cardiovascular parameter values (all originating from the clinical acquisitions) are defined in Supplementary Table 1.

For each subgroup, a baseline virtual subject was created to match the mean of the clinical characteristics. The independent influence of certain cardiovascular properties was then assessed by creating a set of virtual subjects for each defined property, varying that specific entity within the clinical subgroup min-max range, whilst keeping the rest of the properties at baseline level. By doing so, the isolated influence on aortic relative pressure from SV, heart rate, arterial peripheral resistance (calculated from each patient’s mean blood pressure and cardiac output), aortic stiffness, and aortic volumetric size was evaluated. Similarly, virtual flow profiles were generated based on through-plane LVOT flows from the 4D flow MRI of each subgroup, respectively. For each virtual subject, aortic relative pressure was calculated from the LVOT to the end of the thoracic aorta, with relative pressure normalized by aortic length to match the derived clinical data. Note that the LVOT level was assigned at the aortic root, with no valvular influence included in the simulated output (assuming normal valvular function for the entire virtual cohort).

General cardiovascular parameter values, which were kept constant over all virtual subjects, were taken from Willemet et al^18^ and modified when appropriate. The parameters and their corresponding values were: blood density (1050 kg/m^3^), blood viscosity (0.0025 Pa⋅s), velocity profile coefficient (1.33, corresponding to a Poiseuille flow), and outflow pressure (0 Pa).

Aortic stiffness was calculated ensuring that PWV in the virtual subjects was equal to the mean PWV values extracted from each clinical subgroup. With the exact length of the clinical subject aortas unknown, virtual aortic lengths were taken from Willemet et al^18^. Aortic diastolic areas were also extracted from the same reference and scaled according to the ratio of the clinical to virtual average aortic volumetric sizes.

For each virtual subgroup, mean values of left-ventricular ejection time (LVET) and time-to-peak flow were calculated from the corresponding clinical subgroup flow data. A spline interpolation was performed to smooth each flow waveform. LVET was then extracted using the algorithm proposed by Mariscal-Harana et al^[[1]](#footnote-1)^. Time-to-peak flow was extracted as the time of maximum flow. For each subgroup, the average values of LVET and time-to-peak flow were used to generate the virtual flow profiles.

1D simulations were run for a total of 20 s with a 0.1 ms time step to ensure numerical convergence. A visual check of all virtual pressure waveforms at the ascending and abdominal aorta was additionally employed to confirm convergence.

***Supplementary results***

Correlation analysis

Pearson correlation coefficients for a few maximum relative pressure, minimum relative pressure, time-to-peak relative pressure, pulse wave velocity, and aortic stiffness are all provided in Supplementary Table 2. Condensed key results are provided in the main manuscript, however for completeness here follows a detailed description of the obtained correlation results:

For the aortic maximum relative pressure, no significant correlation could be identified with any of the subject characteristics in the DCM cohort.

For the aortic minimum relative pressure, similar behavior could be observed: for the DCM patients no correlations could be inferred with any of the evaluated clinical parameters. For the control group however, slight negative correlation could be inferred between minimum aortic relative pressure and body surface area (R=-0.73, p=0.002).

For TTP no correlation could be inferred for any of the clinical groups at the set significance level.

Lastly, for PWV and derived arterial stiffness, only body mass index was correlated to arterial, stiffness (R=0.755, p = 0.001), however only inferable for the control group.

As noted in the main manuscript, absolute systolic, diastolic, and central blood pressure (SBP, DBP or CBP) did not correlate with any of the evaluated aortic relative pressure metrics.

***Supplementary tables***

**Supplementary Table 1: Parameter variation for virtual cohort.** Parameter variations for each virtual cohort with all data derived from the *in-vivo* imaging. For each subgroup, the baseline virtual subject corresponded to the mean parameter value. Each additional virtual subject within that subgroup corresponded to the variation of a single, isolated parameter (varied from minimum to maximum, one at a time).

| **CV parameters** | **Virtual DCM*_red_*** | | | **Virtual DCM*_pres_*** | | | **Virtual controls** | | |
| --- | --- | --- | --- | --- | --- | --- | --- | --- | --- |
|  | ***min*** | ***baseline*** | ***max*** | ***min*** | ***baseline*** | ***max*** | ***min*** | ***baseline*** | ***max*** |
| **Peripheral resistance,  [mmHg s/mL]** | 0.64 | 0.89 | 1.20 | 0.69 | 0.77 | 0.84 | 0.61 | 0.78 | 1.04 |
| **Stroke volume, [mL]** | 55.0 | 95.5 | 132.0 | 53.0 | 86.0 | 100.0 | 66.3 | 91.7 | 122.9 |
| **Cardiac period, [s]** | 0.75 | 0.91 | 1.13 | 0.59 | 0.80 | 1.00 | 0.70 | 0.88 | 1.03 |
| **Aortic volumetric size, [cm^3^]** | 111.92 | 183.63 | 282.33 | 65.36 | 126.44 | 173.73 | 88.25 | 141.74 | 220.41 |
| **Aortic PWV, [m/s]** | 6.10 | 11.00 | 19.40 | 5.20 | 7.70 | 10.00 | 4.50 | 7.50 | 13.90 |

**Supplementary Table 2: Complete Pearson correlation coefficient table.** Pearson correlation coefficient, for the evaluation of aortic relative pressure measurements. Correlation is evaluated against maximum relative pressure, minimum relative pressure, time to peak relative pressure, pulse wave velocity, and aortic stiffness, respectively. Data is also separated for DCM with reduced LV systolic function (n=8), DCM with preserved systolic LV function (n=5), and control group (n=16), respectively. If a correlation is statistically significant with Bonferroni correction (|R| > 0.5 and p < 0.002, m=21) this is indicated by * (correlations removed by Bonferroni correction indicated by ^+^). Significance is also indicated by the color coding.


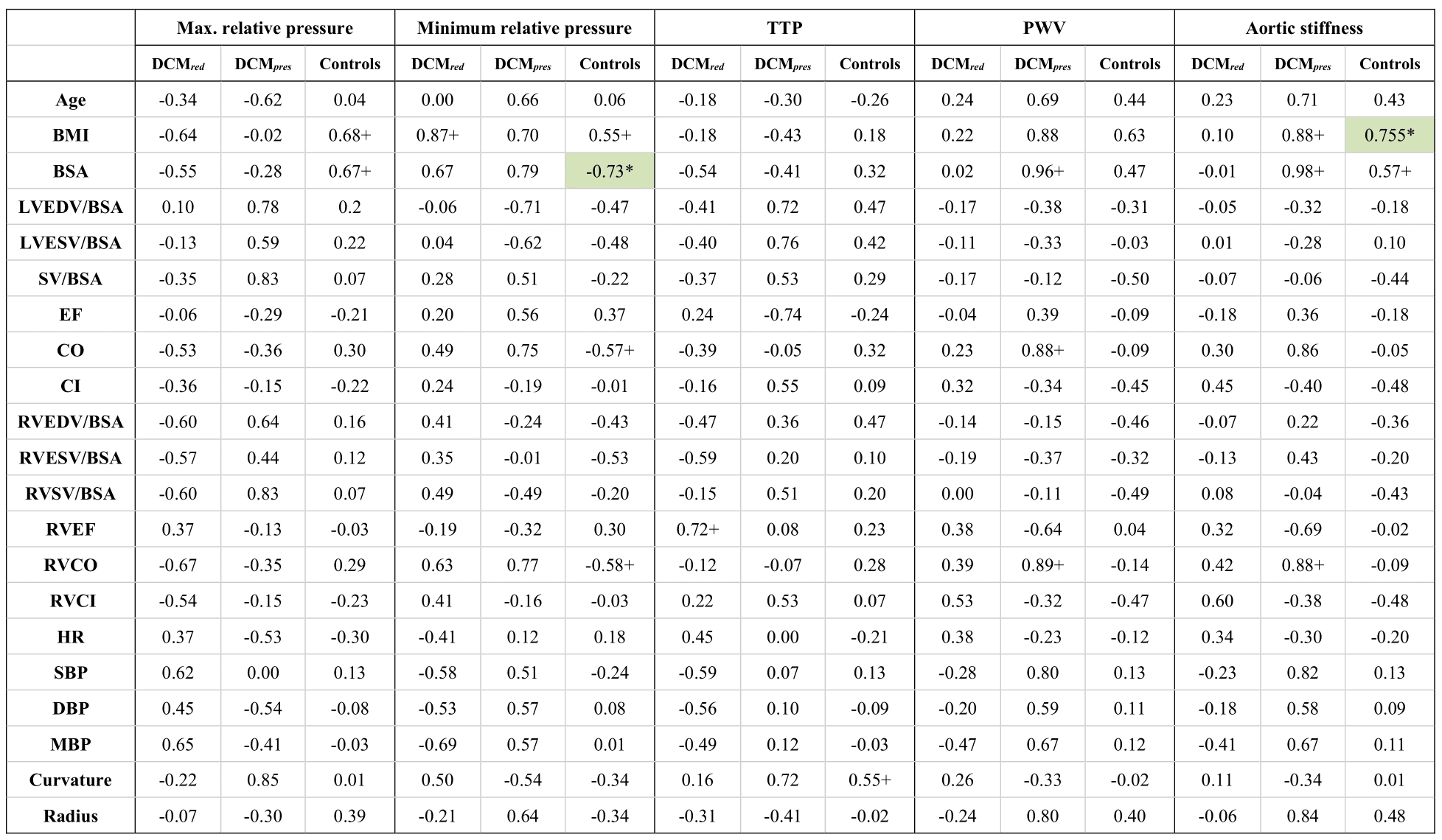


**Supplementary Table 3: Maximum relative pressure variations from the virtual cohort analysis.** Results from the virtual cohort analysis, showing variations in aortic maximum relative pressure per unit length as a function of isolated variations of peripheral resistance, stroke volume, cardiac period, aortic volumetric size, and aortic stiffness, respectively. Data provided for baseline, minimum and maximum ranges, with all values reported in mmHg/m. The bottom row shows the complete range of aortic maximum relative pressure over all variations.

| **Variation variable** | **Virtual DCM*_red_*** | | | **Virtual DCM*_pres_*** | | | **Virtual controls** | | |
| --- | --- | --- | --- | --- | --- | --- | --- | --- | --- |
|  | ***min*** | ***baseline*** | ***max*** | ***min*** | ***baseline*** | ***max*** | ***min*** | ***baseline*** | ***max*** |
| **Peripheral resistance** | 27.5 | 28.6 | 29.4 | 33.0 | 33.5 | 33.9 | 42.7 | 45.1 | 46.9 |
| **Stroke Volume** | 17.2 | 28.6 | 37.9 | 22.1 | 33.5 | 37.8 | 34.2 | 45.1 | 57.2 |
| **Cardiac period** | 28.0 | 28.6 | 29.1 | 32.1 | 33.5 | 34.3 | 43.2 | 45.1 | 46.2 |
| **Aortic volumetric size** | 20.1 | 28.6 | 44.3 | 25.7 | 33.5 | 68.0 | 32.6 | 45.1 | 64.3 |
| **Aortic stiffness** | 21.9 | 28.6 | 29.5 | 27.4 | 33.5 | 38.0 | 34.3 | 45.1 | 52.0 |
| **Total range** | 17.2 | 28.6 | 44.3 | 22.1 | 33.5 | 68.0 | 32.6 | 45.1 | 64.3 |

**Supplementary Table 4: Minimum relative pressure variations from the virtual cohort analysis.** Results from the virtual cohort analysis, showing variations in aortic minimum relative pressure per unit length as a function of isolated variations of peripheral resistance, stroke volume, cardiac period, aortic volumetric size, and aortic stiffness, respectively. Data provided for baseline, minimum and maximum ranges, with all values reported in mmHg/m. The bottom row shows the complete range of aortic maximum relative pressure over all variations.

| **Variation variable** | **Virtual DCM*_red_*** | | | **Virtual DCM*_pres_*** | | | **Virtual controls** | | |
| --- | --- | --- | --- | --- | --- | --- | --- | --- | --- |
|  | ***min*** | ***baseline*** | ***max*** | ***min*** | ***baseline*** | ***max*** | ***min*** | ***baseline*** | ***max*** |
| **Peripheral resistance** | -19.7 | -19.5 | -19.0 | -26.1 | -25.9 | -25.7 | -21.5 | -20.8 | -19.9 |
| **Stroke Volume** | -24.9 | -19.5 | -12.3 | -28.4 | -25.9 | -18.4 | -25.7 | -20.8 | -16.2 |
| **Cardiac period** | -19.9 | -19.5 | -19.1 | -26.9 | -25.9 | -24.2 | -21.2 | -20.8 | -20.1 |
| **Aortic volumetric size** | -32.2 | -19.5 | -12.7 | -24.9 | -25.9 | -19.1 | -36.2 | -20.8 | -20.6 |
| **Aortic stiffness** | -22.3 | -19.5 | -14.2 | -29.2 | -25.9 | -22.2 | -28.2 | -27.8 | -27.2 |
| **Total range** | -32.2 | -19.5 | -12.3 | -29.2 | -25.9 | -18.4 | -36.2 | -20.8 | -16.2 |

1. Mariscal-Harana J, Charlton PH, Vennin S, Aramburu J, Florkow MC, van Engelen A, Schneider T, de Bliek H, Ruijsink B, Valverde I, Beerbaum P, Grotenhuis H, Charakida M, Chowienczyk P, Sherwin SJ, Alastruey J. Estimating central blood pressure from aortic flow: development and assessment of algorithms. *American Journal of Physiology-Heart and Circulatory Physiology*. 2021;320(2):H494–H510. [↑](#footnote-ref-1)
